# Gestational Trophoblastic Neoplasia With Urinary System Metastasis: A Single Center Experience

**DOI:** 10.3389/fonc.2020.01208

**Published:** 2020-07-08

**Authors:** Hongyan Cheng, Junjun Yang, Tong Ren, Jun Zhao, Fengzhi Feng, Xirun Wan, Yang Xiang

**Affiliations:** Department of Obstetrics and Gynecology, Peking Union Medical College Hospital, Chinese Academy of Medical Sciences and Peking Union Medical College, Beijing, China

**Keywords:** gestational trophoblastic neoplasm, neoplasm metastasis, urologic neoplasm, treatment outcome, retrospective study

## Abstract

**Background:** Gestational trophoblastic neoplasia (GTN) with urinary system metastasis is rare. There is limited information about this situation. This study aimed to analyze clinical features, prognostic factors, and survival outcomes of patients with metastasis to the urinary system arising from GTN.

**Methods:** Medical records of 53 consecutive GTN patients with urinary system metastases and treated at Peking Union Medical College Hospital (PUMCH) between 1990 and 2018 were reviewed. The Kaplan-Meier survival analysis was used to describe the overall survival. Prognostic factors were identified using univariate and multivariate analyses.

**Results:** Fifty-three GTN patients with urinary tract metastasis were identified in our institution. The mean age of patients was 30.8 years (range, 23–53 years). Thirty-six (67.9%) patients achieved complete remission (CR), and the remaining 17 (32.1%) showed progressive disease. The 5-year overall survival rate of the entire cohort was 78.4%. Age ≥ 40 years was an independent risk factor for prognosis (HR 12.353, 95% CI 2.203-69.261, *P* = 0.004). Previous failed chemotherapy history (*P* = 0.040) and the presence of brain and/or liver metastases (*P* = 0.024) significantly influenced the survival of GTN patients with urinary tract system metastasis.

**Conclusion:** GTN with urinary tract metastasis is a rare condition. Patients with different metastatic sites have different CR rates and prognosis. Therefore, individualized strategies should be considered for patients with different metastatic sites. Urinary system metastasis is probably not a prognostic factor in GTN patients. Patients aged ≥40, those who had previous failed multidrug chemotherapy, and presented brain and/or liver metastases showed a significant adverse outcome.

## Introduction

Gestational trophoblastic neoplasia (GTN) arises from the abnormal proliferation of placental trophoblastic cells and comprises a malignant invasive mole, choriocarcinoma, placental site trophoblastic tumor, and an epithelioid trophoblastic tumor (1, 2). Patients with GTN often have a significantly increased serum beta-human chorionic gonadotropin (β-HCG) concentration which can therefore be used as a tumor marker. The International Federation of Gynecology and Obstetrics (FIGO) scoring system was used to predict the prognosis of GTN patients. According to FIGO Cancer Report 2018, GTN patients were divided into a low-risk group (FIGO score <7), high-risk group (7 ≤ FIGO score ≤ 12), and an ultra-high-risk subgroup (FIGO score > 12) (1). Although the cure rates can reach 100% in low-risk and to >90% in high-risk GTN patients, the prognosis of ultra-high-risk GTN and refractory GTN patients is still poor (2–4), especially of patients with liver (5) or brain (6) metastases.

GTN with urinary system metastasis is rare. The incidence of renal metastasis reported varies from 1.3% (7) to 6.9% (8) patients in the literature. As for GTN metastasis to the bladder and ureter, only a few cases have been reported (9–12). Initial symptoms of GTN with urinary system metastasis can be backache, hematuria, anuria, uronephrosis, renal mass, or spontaneous renal hemorrhage (13). Due to limited information about GTN with urinary system metastasis, we conducted a retrospective analysis to evaluate the clinical features, prognostic factors, and survival outcomes of GTN patients with urinary system metastasis.

## Materials and Methods

### Patients

This study is a retrospective study and was conducted between January 1990 and December 2018 at Peking Union Medical College Hospital (PUMCH). The follow-up time was defined until August 2019. Medical records were obtained from patients' admission and discharge files. Patients were excluded if they had another concurrent malignancy. Urinary system metastatic disease was diagnosed based on GTN history and lesions located in the kidneys, ureter, and bladder, which were found on ultrasonography, computed tomography (CT), or magnetic resonance imaging (MRI). This study was approved by The Ethics Committee of PUMCH review board. The patients/participants provided their written informed consent to participate in this study. Written informed consent was obtained from the individual(s) for the publication of any potentially identifiable images or data included in this article.

### Treatment

All GTN patients with urinary system metastasis underwent evaluation before treatment, which included medical history, physical examination, routine blood test, biochemistry and serum β-HCG level test, ultrasonography, and CT or MRI scan of the chest and abdomen. The FIGO 2000 staging and score system was used to identify the disease stage and score.

Based on previous chemotherapy that the patients received, several different chemotherapy regimens were used: the FAEV (fluorouracil/floxuridine, actinomycin-D, etoposide, vincristine) or FAV (fluorouracil/floxuridine, actinomycin-D, vincristine) regimen was used for patients who did not previously receive the fluorouracil/floxuridine-based combination chemotherapy (14, 15). If the serum β-HCG level presented an unsatisfactory decline or the patient developed drug resistance, the EMA/CO (etoposide, methotrexate, actinomycin-D/cyclophosphamide, vincristine), EMA/EP (etoposide, methotrexate, actinomycin-D/etoposide, cisplatin), and TE/TP (paclitaxel, etoposide/paclitaxel, cisplatin) regimens were used as the replacement (1). In addition to systemic chemotherapy, GTN patients with brain metastasis received an intrathecal injection of methotrexate, and patients with bladder metastasis received fluorouracil bladder perfusion (250 mg every other day, four times for one cycle). Furthermore, patients also received an additional two to four courses of consolidation systemic chemotherapy after the serum β-HCG level was normal.

### Evaluation

Complete remission (CR) was defined as normal serum β-HCG levels measured for 3 consecutive weeks. Resistance was defined as a plateau or increased serum β-HCG level after at least two courses of chemotherapy. Relapse was defined as an increase in the serum β-HCG levels, again, 1 month after CR.

### Statistical Analysis

A statistical analysis was performed using SPSS software version 19 (IBM Company, Armonk, NY, USA). The Fisher's exact test was used for categorical variables. The Kaplan-Meier survival analysis and a log-rank test were used to describe overall survival (OS). Multivariate analysis was performed using the Cox proportional regression model. *P*-value of < 0.05 was considered statistically significant.

## Results

### Clinical Characteristics

Between 1990 and 2018, 3,172 patients were diagnosed with GTN in total, of which 53 (1.67%) patients presented metastasis to the urinary system in the PUMCH. Detailed clinical characteristics are shown in Table 1. The mean age of patients was 30.8 years (range, 23-53 years). Patients' complaints included hematuria (11 patients), backpain (4 patients), uronephrosis (4 patients), and renal hemorrhage (1 patient), another 23 patients were found to have bladder or kidney metastases upon imaging examination. Among the 53 patients, 24 (45.3%) received primary treatment in our institution, and the remaining 29 (54.7%) had previous failed chemotherapy elsewhere and then referred to our hospital. The median pretreatment serum β-HCG levels in patients were 186,659 IU/L (range, 11.2-3776700 IU/L). According to the FIGO 2000 staging score system, (16) the median score was 13 (range, 3–20).

**Table 1:** Clinical characteristics of GTN patients with urinary system metastasis (*n* = 53).

**Characteristics**		**No. of patients (%)**
Age (years)	<40	48 (90.5)
	≥40	5 (9.5)
Histology	IM	5 (9.5)
	CC	48 (90.5)
Antecedent pregnancy	Mole	15 (28.3)
	Miscarriage	20 (37.7)
	Term	18 (34.0)
Interval from index pregnancy, months	<4	15 (28.3)
	4-6	7 (13.2)
	7-12	8 (15.1)
	≥12	23 (43.4)
Previous failed chemotherapy	No	24 (45.3)
	Single drug	3 (5.6)
	Two or more drugs	26 (49.1)
Pretreatment β-HCG, IU/L	<10^3^	5 (9.5)
	10^3^-10^4^	5 (9.5)
	10^4^-10^5^	15 (28.3)
	>10^5^	28 (52.7)
Urinary system metastasis	Kidney	34 (64.2)
	Kidney + bladder	2 (3.7)
	Bladder	9 (17.0)
	Bladder + ureter	3 (5.6)
	Ureter	5 (9.5)
Lung metastasis	Yes	45 (84.9)
	No	8 (15.1)
Site of metastases (lung excluded)	Urinary system + liver	3 (5.6)
	Urinary system + brain	14 (26.4)
	Urinary system + liver + brain	6 (11.3)
	Urinary system + other site^a^	6 (11.3)
	Only urinary system	24 (45.3)
FIGO score	<7	6 (11.3)
	7–12	19 (35.9)
	>12	28 (52.8)

a *Including the gastrointestinal tract, spleen, pancreas, adrenal glands, skin and bone*.

*GTN, gestational trophoblastic neoplasia; IM, invasive mole; CC, choriocarcinoma; HCG, human chorionic gonadotropin; FIGO, International Federation of Gynecology and Obstetrics*.

Among the 53 patients with urinary metastases, 29 of them had combined other site metastases, and 24 patients had solitary urinary tract metastases. In total, there were 34 GTN patients (64.2%) with renal metastasis, nine (17.0%) with bladder metastasis, five (9.5%) with ureter metastasis, two (3.7%) with both kidney and bladder metastases disease, and the remaining three (9.5%) had both bladder and ureter metastases disease. In addition to urinary system metastases, the distant metastatic sites included the lung (45 patients); brain (14 patients); liver (three patients); both brain and liver (six patients); and unusual sites such as the gastrointestinal tract, spleen, adrenal gland, pancreas, skin, and bone.

### Treatment

In this study, the mean chemotherapy number of cycles that the patients received was 11 (range, 2–40 cycles). Among the 53 GTN patients with urinary tract system metastases, 24 received the FAEV/FAV regimen as the first-line multiagent chemotherapy, and the remaining 29 patients who were transferred from other hospitals with a history of failed chemotherapy were treated with salvage chemotherapy, including FAEV/FAV, EMA/CO, EMA/EP, and TE/TP. The mean chemotherapy number of cycles for patients with solitary urinary tract metastases and patients with combined other site metastases were 10.6 (range, 4 to 26) and 13.8 (2–40), respectively. There was no difference in the type of chemotherapy patients received and the number of chemotherapy courses between the two groups. In addition, three patients died during the salvage chemotherapy treatment process. There were 39 patients who received surgical treatment, of whom 16 underwent hysterectomy, five underwent pulmonary lobectomy, and eight underwent craniotomy.

Additionally, for urinary system metastatic lesions, there were six patients with bladder metastasis who received floxuridine bladder perfusion, three of whom underwent excision of the bladder lesions. All of them showed a satisfactory response. For three patients with ureteral metastasis, double-J ureteral stenting was performed to relieve the ureteral obstruction. Another two patients with renal metastasis underwent unilateral nephrectomy due to an isolated metastatic lesion that was non-responsive to chemotherapy.

### Outcome and Prognosis

Among the 53 patients, 36 (67.9%) achieved CR and the remaining 17 (32.1%) showed disease progression. Three patients died of disease progression during the treatment process, and six patients died during the follow-up period. Among the 23 patients with brain and/or liver metastasis, the CR rate was only 52.2%, which was remarkably lower than that in patients without brain and/or liver metastasis (52.2% vs. 80%, *P* = 0.041). Furthermore, the difference in CR rates between the age groups of <40 and ≥40 years (75 vs. 0%, *P* = 0.002) and between patients with and without previous failed multidrug chemotherapy (53.8 vs. 81.5%, *P* = 0.042) was significant (Table 2). For patients with urinary system metastasis, the CR rate of patients with renal metastasis was lower than that of patients with other urinary system metastasis (58.3 vs. 88.2%), but without statistical significance (*P* = 0.056).

**Table 2:** Comparison of CR rate of 53 GTN patients with urinary system metastasis for age, previous failed multidrug chemotherapy and with liver/brain metastasis.

	**No. of CR**	**No. of non-CR**	**CR rate**	***P*-value**
Age				0.002^a^
<40	36	12	75.0%	
≥40	0	5	0.0%	
Previous failed multidrug chemotherapy				0.042^a^
Yes	14	12	53.8%	
No	22	5	81.5%	
Brain and/or liver metastasis				0.041^a^
Yes	12	11	52.2%	
No	24	6	80.0%	

a *P < 0.05 indicated statistically significant differences*.

*CR, complete remission; GTN, gestational trophoblastic neoplasia; No., Number*.

**Figure 1:**
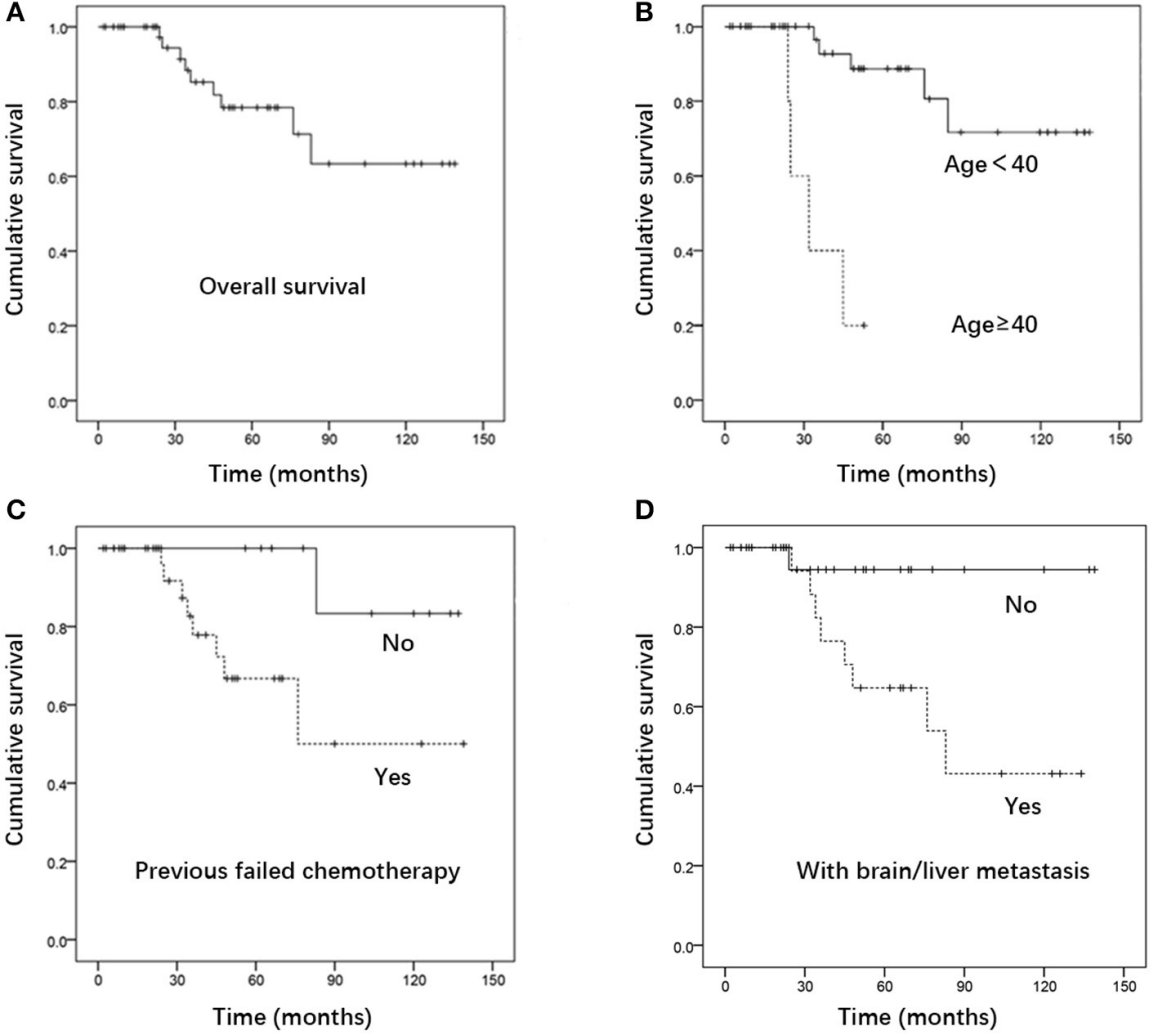
Kaplan- Meier curve for **(A)** overall survival. **(B)** Survival of patients aged ≥40 Vs. <40, **(C)** survival of patients with Vs. without previous failed chemotherapy, **(D)** survival of patients with Vs. without brain and/or liver metastases (*n* = 53).

The median follow-up time was 36 months (range, 6–139 months). The estimated 5-year overall survival rate of the entire cohort was 78.4%. Patients in the GTN group who presented with urinary tract system metastasis, without brain and/or liver metastases, had a favorable 5-year overall survival and CR rate, reaching 94.4 and 80%, respectively. This result suggests that urinary system metastasis is probably not a prognostic factor in GTN patients. The results revealed that patients aged ≥40 years (*P* < 0.001), those with a history of previous failed chemotherapy (*P* = 0.040), and those with brain and/or liver metastases (*P* = 0.024) had a significantly poorer survival rate (Figure 1). Multivariate analysis showed that being ≥ 40 years of age was an independent risk factor for outcome (HR 12.353, 95% CI 2.203-69.261; *P* = 0.004) (Table 3).

**Table 3:** The univariate and multivariate analysis of prognostic risk factors on GTN patients with urinary system metastasis (*n* = 53).

**Characteristics**	***P-*****value (HR, 95% CI)**	
	**Univariate analysis**	**Multivariate analysis**
Age (years)		
<40	-	1
≥40	<0.001^*^	0.004^*^ (12.353, 2.203–69.261)
Antecedent pregnancy		
Mole	-	1
Non-molar pregnancy	0.052	0.059 (21.279, 0.425–1064.453)
Interval from index pregnancy, months		
<12	-	1
≥12	0.126	0.560 (2.192, 0.309–15.567)
Previous failed chemotherapy		
No	-	1
Yes	0.040^*^	0.084 (2.062, 0.030–4.130)
Pretreatment β-HCG, IU/L		
≤ 10^5^	-	1
>10^5^	0.760	0.165 (0.206, 0.020–2.178)
Brain and/or liver metastasis		
No	-	1
Yes	0.024^*^	0.693 (0.548, 0.028–10.898)
Surgical procedures		
Yes	-	1
No	0.059	0.478 (1.228, 0.089–16.989)
Figo score		
≤ 12	-	1
>12	0.210	0.761 (0.315, 0.021–4.766)

* * P <0.05 indicated statistically significant differences*.

*HR, hazard ratio. FIGO, International Federation of Gynecology and Obstetrics*.

## Discussion

The incidence of GTN with urinary system metastasis reported in the literature is 1–14% (7, 17). In our study, GTN patients with urinary tract metastasis accounted for 1.67% of all GTN patients between 1990 and 2018. The overall CR rate was 67.9%, and the 5-year overall survival rate was 78.4%. Patients in the GTN group who presented urinary tract system metastasis without brain and/or liver metastases, had a favorable 5-year overall survival and CR rate, reaching 94.4 and 80%, respectively. This result suggests that urinary system metastasis is probably not a prognostic factor in GTN patients. However, due to the rarity of the stage IV disease, it is difficult to match the proper control group to evaluate the contribution of solitary urinary tract (or with only lung) metastases on CR rate and prognosis. Nonetheless, metastatic lesions located in the urinary tract may influence the efficiency of chemotherapeutic agents because most of them are metabolized by the kidney and bladder. Treatment with a chemotherapeutic agents increases the likelihood of renal dysfunction, due to the inevitable urinary excretion of various chemotherapeutic metabolites that can cause nephrotoxic effects (18). A study showed that patients with metastatic kidney cancer have worse central hemodynamic measurements (19). In addition, impaired renal excretion and decreased metabolism may lead to systemic toxicity, such as myelosuppression (20).

**Figure 2:**
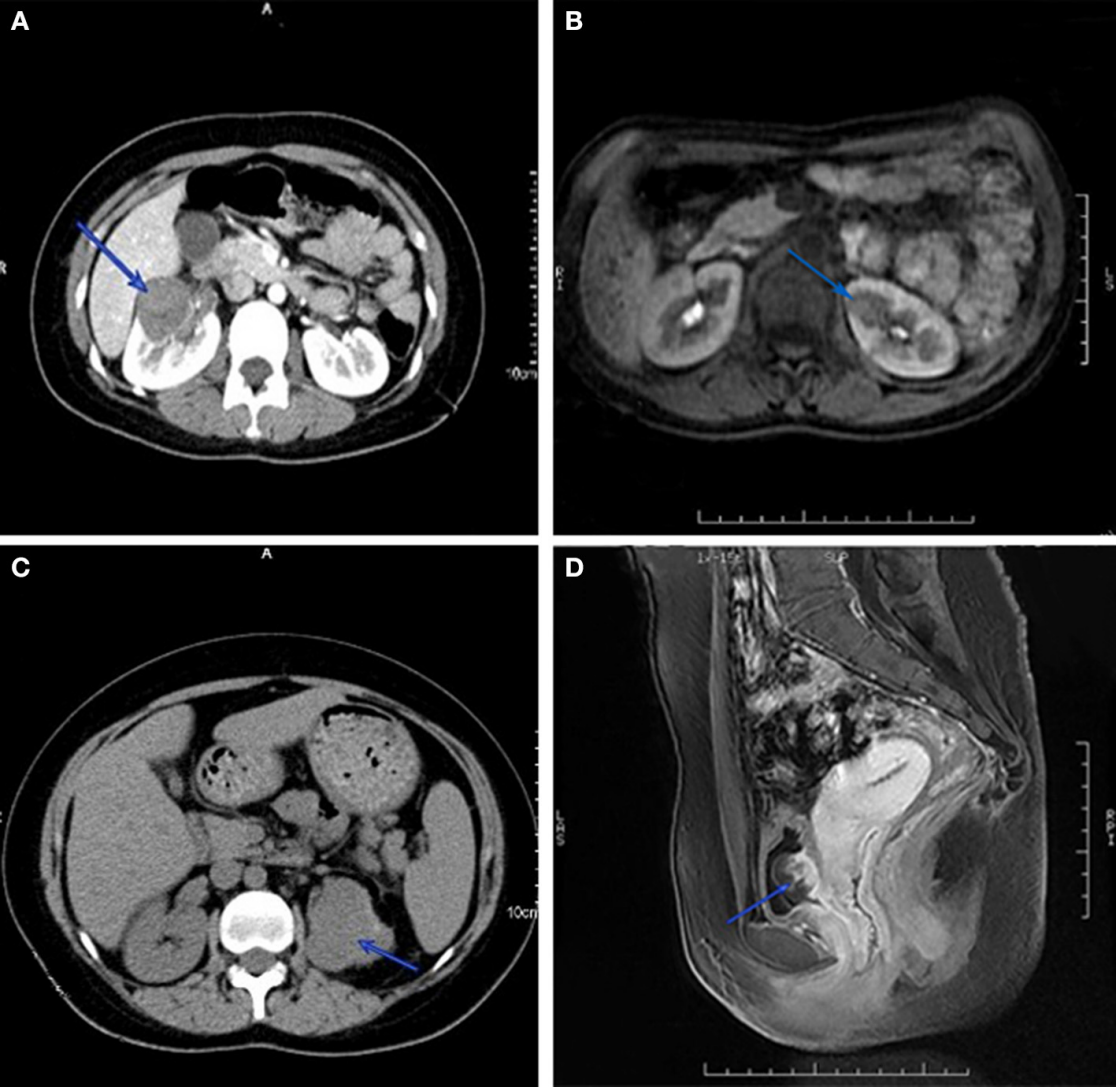
Computed tomography imaging of GTN patients with renal metastasis. Isolated renal mass in right **(A)** and left kidney **(B)**, and multiple lesions disform the left kidney **(C)**. Magnetic resonance imaging of metastatic lesion in bladder **(D)**.

Intravenous chemotherapy is a common treatment for GTN patients with urinary system metastasis in clinical practice. Nevertheless, individualized strategies should be considered for patients with different metastatic sites. Regarding renal metastasis, chemotherapy can reduce or eliminate lesions in most patients. A few patients with renal lesions that were unresponsive to chemotherapy had their kidney lesion excised in the presence of an isolated renal mass (Figures 2A,B), and unilateral nephrectomy was applicable when multiple metastatic lesions were present, causing loss of the normal shape of the kidney (Figure 2C). In a small number of cases, when GTN patients present spontaneous renal hemorrhage, angioembolization can be performed for hemorrhage control (13). In this study, two patients underwent unilateral nephrectomy and one underwent renal artery embolization for bleeding control. In both cases, patients demonstrated a favorable outcome. Data on bladder and ureter metastases have not previously been reported in the literature. From experience in our institution, fluorouracil bladder perfusion was effective for bladder metastasis. Concerning large metastatic lesions in the bladder and ureter (Figure 2D), chemotherapy can shrink and soften the lesion-surrounding tissue, which is beneficial for ureteral separation during surgery. For patients with hydronephrosis and/or ureterectasis, imaging such as intravenous urography or CT can determine whether the hydronephrosis and/or ureterectasis is caused by tumor metastasis or extrinsic pression. Double-J ureteral stenting is recommended for those patients to dilate the ureter and alleviate the hydronephrosis, then chemotherapy should start as soon as possible to salvage the affected ureter and kidney. For patients suffering double-J stent failure, surgery is considered to remove the metastatic lesion and part of the ureter, then ureteroneocystostomy or ureteroneocystostomy can be performed.

Our data demonstrated that GTN patients who presented brain and/or liver metastases have a poor outcome. It is worth noting that all patients with renal metastasis in this group reached a lower CR rate and higher overall survival rate. However, for GTN patients with only bladder and/or ureteral metastasis, the outcome and CR rate were both favorable. Therefore, clinical practitioners should be more attentive to patients with renal metastasis. Additionally, results showed that patients with an age of ≥40 years with previous failed chemotherapy history have an adverse outcome, which is similar to our previous studies' findings (2, 5, 6). There were only five patients aged 40 or older in our study, and despite the fact that the conclusion is probably biased, a small sample size is an inevitable limitation of a rare disease.

## Conclusions

Although this study has a retrospective limitation and relatively small sample size, our findings represent the largest cohort of GTN patients with urinary system metastases and provide unique information for the clinical treatment of these patients. In conclusion, GTN with urinary tract metastasis is a rare condition. Patients with different metastatic sites have different CR rates and prognosis. Therefore, individualized strategies should be considered for patients with different metastatic sites. Urinary system metastasis is probably not a prognostic factor in GTN patients. Patients aged ≥40, those who had previous failed multidrug chemotherapy, and presented brain and/or liver metastases, showed a significant adverse outcome.

## Data Availability Statement

The raw data supporting the conclusions of this article will be made available by the authors, without undue reservation.

## Ethics Statement

The studies involving human participants were reviewed and approved by The Ethic Committee of Peking Union Medical College Hospital. The patients/participants provided their written informed consent to participate in this study. Written informed consent was obtained from the individual(s) for the publication of any potentially identifiable images or data included in this article.

## Author Contributions

Study conception and design: YX and JY. Literature review and data extraction: HC and JY. Quality control: JZ and TR. Statistical analysis: FF and XW. Manuscript preparation: HC and JY. Manuscript review: JZ, TR, FF, XW, and YX. All authors contributed to the article and approved the submitted version.

## Conflict of Interest

The authors declare that the research was conducted in the absence of any commercial or financial relationships that could be construed as a potential conflict of interest.
